# Thyroid Cancer in Childhood Leukemia Survivors: A Systematic Review of the Incidence and Survival Outcomes

**DOI:** 10.3390/jcm14124248

**Published:** 2025-06-14

**Authors:** Vasiliki Rengina Tsinopoulou, Eleni P. Kotanidou, Savvas Kolanis, Athanasios Tragiannidis, Emmanouel Hatzipantelis, Assimina Galli-Tsinopoulou

**Affiliations:** 2nd Department of Paediatrics, School of Medicine, Faculty of Health Sciences, Aristotle University of Thessaloniki, AHEPA University Hospital, 54636 Thessaloniki, Greece; vitsinop@auth.gr (V.R.T.); epkotanidou@auth.gr (E.P.K.); savvasks@auth.gr (S.K.); atragian@auth.gr (A.T.); hatzip@auth.gr (E.H.)

**Keywords:** acute lymphoblastic leukemia, thyroid carcinoma, event-free survival, radiotherapy, second malignant neoplasm, second primary cancer

## Abstract

**Background/Objective:** Radiotherapy for leukemia, the most common childhood malignancy, often exposes patients to radiation, increasing the risk of second malignancies, including thyroid cancer. To assess the incidence and survival outcomes of thyroid cancer after childhood acute lymphoblastic leukemia (ALL). **Methods**: We systematically reviewed articles reporting the incidence of thyroid cancer in childhood leukemia survivors (age at diagnosis < 18 years) published between 2000–2024 in Science Direct, PubMed, Google Scholar, CENTRAL, and EMBASE. The Newcastle Ottawa Scale was utilized to appraise the methodological quality of the included studies. Descriptive statistics and calculations of incidence were performed using Microsoft Excel. **Results**: The literature search yielded 1265 articles, of which 18 met the inclusion criteria. Data from 135,861 childhood cancer survivors, among whom 102,070 had a confirmed diagnosis of childhood leukemia, including ALL. The crude incidence of secondary malignancies after childhood leukemia was 10.1 per 1000 patients. Among these, 1.5 per 1000 patients developed second thyroid carcinomas. Overall, 14.6% of the second malignancies in childhood leukemia survivors were thyroid carcinomas, mostly of the papillary type. Survival rates after second thyroid cancer were 100% in all 11/18 studies reporting this outcome. Radiotherapy had been used as part of ALL treatments in 17/18 studies. The use of radiotherapy, female sex, and younger age at the diagnosis of primary ALL emerged as important risk factors for thyroid cancer. **Conclusions**: Thyroid carcinomas account for ~15% of secondary malignancies after childhood leukemia, with radiation remaining a significant risk factor despite its overall reduced use for the treatment of ALL in the last few decades. Importantly, survival rates remain high. Further research is warranted to determine the incidence and outcomes of thyroid cancer in childhood ALL survivors

## 1. Introduction

Leukemia is a common malignancy in pediatric populations, accounting for a significant percentage of cancer-related mortality and morbidity in this population [1]. The two main phenotypes of leukemia, acute myelogenous leukemia (AML) and acute lymphoblastic leukemia (ALL), account for about 30% of all pediatric leukemia cases. Among these two phenotypes, ALL is more prevalent than AML in the pediatric population, accounting for 80% of childhood leukemia occurring under 19 years of age, whereas AML only represents 15–20% [1,2]. Other types of leukemia, such as chronic myeloid leukemia (CML), only account for about 3% of all childhood leukemia cases [3]. These leukemia subtypes differ in terms of their etiologies, prognosis, and treatment response, and even in terms of their clinical manifestations. Across all etiologies, both prenatal and postnatal factors play a crucial role in the determination of the onset of childhood leukemia [4].

While leukemia is still a major cause of morbidity and mortality in children, the survival of these patients has greatly improved over the years. Currently, it is estimated that more than 85% of the patients diagnosed with childhood cancer survive for at least 5 years [5]. However, despite being very much desired, this increased survival is accompanied by an increased risk of developing comorbidities related to childhood cancer diagnosis [5], including the development of second primary malignancies (SPMs) after the successful treatment of the childhood cancer. The 30-year cumulative incidence of SPMs in patients with primary leukemia was reported to be 5.6% (95% confidence interval [CI] = 4.5–6.7%) [6]. Risk factors for developing secondary neoplasms after childhood cancer include female sex, age at primary childhood cancer diagnosis, the use of radiation therapy, the intensity of chemotherapeutic agents, hematopoietic stem cell transplantation due to high-dose conditioning regimens, and immunotherapeutic regimens [6,7].

Many radiological and epidemiological studies have established that exposure to radiation during childhood is one of the main risk factors for the development of thyroid cancer after childhood cancer [8]. This is expected considering the high sensitivity of the thyroid gland, especially the pediatric one, to radiation. The use of radiotherapy in ALL has decreased dramatically over the past few decades, mainly owing to the development and intensification of chemotherapy protocols [9]. However, regimens for the treatment of pediatric cancer may still involve the use of radiation, making survivors prone to developing second cancers, including thyroid carcinoma [8]. While a rare occurrence in childhood cancer survivors, thyroid carcinoma still accounts for some of the major cancers after childhood cancer.

This systematic review aimed to summarize the current evidence on the incidence and related outcomes of thyroid carcinoma after childhood leukemia, specifically ALL, given its higher prevalence in the pediatric population.

## 2. Materials and Methods

### 2.1. Protocol and Registration

This systematic review was conducted according to the guidelines of PRISMA 2020 (Preferred Reporting Items for Systematic Reviews and Meta-Analyses) [10]. The protocol of the review was registered in the Open Science Framework (https://doi.org/10.17605/OSF.IO/C4J5R).

### 2.2. Literature Search

The literature search was conducted independently by two reviewers, VRT and AGT, using two approaches. In the first approach, the reviewers used a set of keywords to perform a comprehensive search in five electronic databases, i.e., Science Direct, PubMed, Google Scholar, CENTRAL, and EMBASE, in order to identify articles published between 1 January 2000 and 31 December 2024. The keywords utilized for the electronic search included (Second Malignancy OR Second Neoplasms, OR Second Thyroid Cancer) AND (Pediatric Leukemia OR Childhood Leukemia Survivors OR Childhood Cancer Survivors). These keywords were modified for each database to maximize the number of results. In the second approach, the reviewers checked the lists of the references of the obtained studies to find additional studies that would not have been identified in the initial search. Finally, the reviewers searched for any trial registered in the clinical trial.gov registry for any completed trial with results that did not have published outcomes available.

### 2.3. Eligibility Criteria

After obtaining all the articles from the databases and registries, the authors utilized the prespecified eligibility criteria to analyze each article before including it in the review.

Articles were selected if they met the following inclusion criteria: (1) published in English, (2) inclusion of childhood survivors of primary leukemia (diagnosis of leukemia prior to age of 18 years), (3) reporting the incidence of thyroid cancer after primary leukemia, (4) designed as case-control studies, cohort studies, and case series.

Studies were excluded from the review during the eligibility analysis if they met the following exclusion criteria: (1) not including patients with primary leukemia; (2) including patients diagnosed with leukemia after the age of 18 years; (3) not reporting the incidence of thyroid cancer as a second malignancy after primary leukemia; (4) being secondary studies, including systematic reviews, meta-analyses, and narrative reviews.

### 2.4. Data Extraction

Two reviewers, VRT and AGT, independently conducted the data extraction for the review. In case of disagreement during the data extraction process, the two reviewers discussed the arising discrepancies until they reached a consensus. As per the PRISMA guidelines, before data extraction, the authors conducted a review of all the references obtained through various phases. The initial phases entailed abstract screening for the relevance of the articles, after which all irrelevant articles were eliminated. The full text of the remaining articles was then searched and also assessed for eligibility. Those that met the inclusion criteria were used for data extraction. The information obtained from each article included the author ID, study setting, subtype of leukemia, sample size (overall, leukemia only), age at the diagnosis of primary leukemia, time to the diagnosis of thyroid cancer or SPM, type of treatment for leukemia, type of treatment for thyroid cancer, analyzed outcomes, survival of patients with second thyroid cancer, and follow-up time. The reviewers also obtained details of second malignancies, including the overall incidence of second malignancies and the incidence of thyroid cancer, as well as the type of thyroid cancer, if specified, in the study. Other relevant factors, such as the dose of radiation in cases where radiotherapy was used for the treatment of leukemia, were also extracted.

### 2.5. Quality/Risk-of-Bias Assessment

An assessment of the risk of bias was performed by using the Newcastle Ottawa Scale (NOS). This scale, which is adapted for both cohort and case-control studies, is used to analyze the methodological quality of each study in terms of cohort selection, cohort comparability, and outcome reporting, taking into account the follow-up period. Each reviewer rated the included studies using stars as specified in an accompanying key. The total number of stars for each of the NOS domains was then used to determine the overall Agency for Healthcare Research and Quality standard of the study [11].

### 2.6. Statistical Analysis

Descriptive statistics, the calculations of the means and standard deviations of raw data provided for the subgroups of patients, and the calculation of the crude incidence of thyroid cancer among childhood leukemia survivors were performed using Microsoft Excel version 16.66.1.

## 3. Results

### 3.1. Search Results

**Figure 1:**
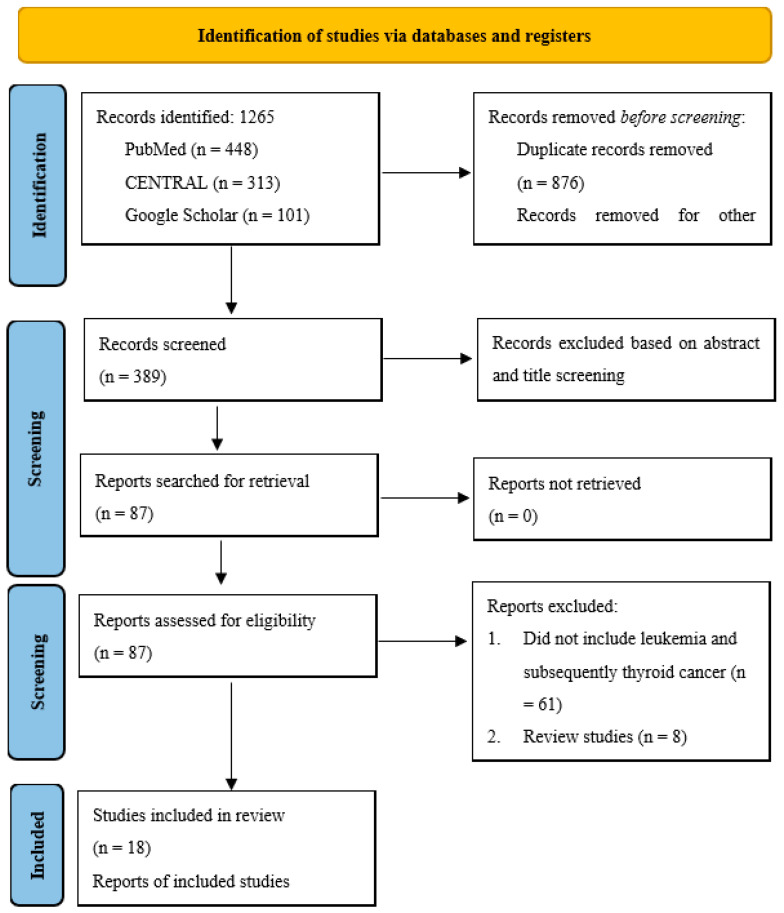
PRISMA diagram summarizing the search strategy of the included studies, along with reasons for excluding studies.

Our literature search yielded 1265 articles, among which 876 were duplicates and thus were excluded from the study. This led to 389 abstracts being screened. After the abstract screening, 302 articles were considered irrelevant to the topic of study and were thus excluded from the study, and the remaining 87 articles were searched for the retrieval of the full text and an assessment for eligibility for our review. After the eligibility assessment, 18 articles were included in the review, and 69 were excluded. The following were the reasons for excluding these 69 studies: 8 were secondary studies, 51 did not report the incidence of thyroid cancer as a second malignancy of leukemia, and 10 were duplicate reports from the same cohort of patients, including the Childhood Cancer Survivor study and the SEER (Surveillance, Epidemiology, and End Results) database. A PRISMA diagram summarizing the search strategy followed is presented in Figure 1.

### 3.2. Characteristics of the Included Studies

The characteristics of the included studies are presented in Table 1. This review included 18 cohort studies that summarized data from a pooled cohort of 135,861 patients, among whom 102,070 had a confirmed diagnosis of leukemia prior to the age of 18 years. The median follow-up time ranged from 0 to 40 years across studies.

Table 2 shows the disease and treatment characteristics. The common types of leukemia were ALL and AML. The age of the patients with thyroid cancer at the time of the diagnosis of leukemia (or other primary cancer, if not specified) ranged from 0 to 19.7 years. The latency to thyroid cancer development after the diagnosis of primary leukemia ranged from 1 to 35.5 years. The different subtypes of thyroid carcinoma reported included papillary and follicular thyroid carcinoma. Most of the included studies analyzed the incidence of second malignancies, including thyroid carcinoma. A few studies analyzed the outcomes after second malignancies, including survival, and a few studies performed a subgroup analysis of these outcomes based on the type of cancer.

### 3.3. Methodological Quality of the Included Studies

All the included studies had good methodological quality in both the selection of participants and the reporting of the outcomes. The comparability of the different cohorts was also good within each study, and most of the studies adjusted the reported outcomes for confounding factors such as age, sex, and the occurrence of death (Table 3).

### 3.4. Incidence of Thyroid Cancer

All the included studies reported the incidence of second malignancies after childhood leukemia, with one of the second malignancies being thyroid carcinoma. In a sample of 102,070 patients with leukemia, including ALL, there were 1108 patients who developed SPMs, of whom 162 developed second thyroid cancer (14.6%). The crude incidence of second malignancies in leukemia survivors was 10.1 malignancies per 1000 patients, while that for second thyroid carcinomas was 1.6 malignancies per 1000 patients. The study by Taylor et al. did not provide the total number of patients with leukemia despite reporting that of patients with leukemia who developed thyroid cancer, and hence, it was excluded from further calculations [27]. Thus, considering 17 studies, the crude incidence of second thyroid carcinomas among patients with childhood leukemia was 1.5 malignancies per 1000 patients. However, there was great heterogeneity across studies, with the effect size (% rate) ranging from 0.045% in the study by Bhatia et al. to 100% in the studies by Achaya et al. and Martucci et al. Removing these two later studies as major outliers did not have a major impact on incidence (1.4 per 1000 persons), as both studies included only a few patients [12,15,20]. The additional removal of three more studies reporting quite high rates of thyroid cancer among childhood leukemia patients (Gow et al.: 35.3%, Koh et al.: 11.1%, Oudin et al.: 52% [18,19,22]) also did not change the crude incidence (1.1 per 1000 patients) significantly.

### 3.5. Risk Factors for the Development of Thyroid Cancer

Most of the included studies did not specifically attempt to determine risk factors for the development of thyroid cancer, specifically, but rather for the development of second malignancies, in general.

Across the studies, the emerging risk for the development of second malignancies was the regimens used to treat the primary malignancies, including radiation, allogenic bone marrow transplantation, and chemotherapy agents, with radiation-based regimens being associated with greater risk than other regimens [26,29]. Seventeen of the eighteen included studies reported the use of radiotherapy to treat leukemia, either as monotherapy or in combination with other regimens (Table 2). Bhatia et al. reported that patients who received 2400 cGY of radiation therapy for primary ALL had a significantly higher risk of developing thyroid cancer than the general population (relative risk of 30.8; 95% CI = 1.2–62.9) [15]. Furthermore, Veiga et al. found that, across all primary tumors, all regimens that included radiation directed to the thyroid (regardless of the dose) significantly increased the risk of thyroid cancer compared to chemotherapy alone [29]. Interestingly, in patients who received radiation doses <2000 cGy, the use of chemotherapy, and particularly of anthracyclines and alkylating agents, was associated with a 4-fold higher risk for thyroid cancer. Similar results were reported by Taylor et al., whereby 88% of the 50 childhood cancer survivors with thyroid SPM included in their study had received radiation directed either within or close to the thyroid gland, with doses ranging from 1000 to 4600 cGy [27]. Of the 50 survivors, 9 had survived childhood ALL and had received radiotherapy to the whole brain, imposing a risk of exposure of the thyroid [27]. Total body irradiation at high doses (≥1000 cGy of singledose or ≥1300 cGy of fractionated) was reported to be associated with a higher risk of solid cancers [26]. In the study by Borgmann et al., SPMs were significantly associated with stem cell transplantation, high cumulative doses of cranial irradiation (>1800 cGy), as well as with etoposide and cyclophosphamide regimens [16]. In contrast, Oudin et al. reported only a trend for the risk imposed by the type of treatment followed for leukemia [22].

The age at childhood leukemia diagnosis also appeared as an important factor for developing SPM. Maule et al. highlighted that patients diagnosed with leukemia at the age of 1–4 years were more prone to developing second malignancies, including thyroid cancer [21]. The authors pointed out that such an increased risk was expected considering that the incidence of pediatric leukemia is highest within that age group [21]. Taylor et al. reported similar results, with all patients with thyroid cancer who were survivors of ALL having been diagnosed with ALL between 2 and 9 years of age [27].

Contrary to other studies, Oudin et al. found that female sex was the only significant risk factor for the development of thyroid cancer after childhood leukemia in a sample of 502 patients [cumulative incidence at 20 years: 17.1% (95% CI = 10.6–27) vs. 2.3% (95% CI = 0.6–8.2) for female and male, respectively, *p* < 0.001] [22]. Across most studies that provided information on the sex, most of the affected patients with SPMs were women (Table 1).

### 3.6. Type and Treatment of Thyroid Cancer

In most cases, the type of thyroid cancer in childhood leukemia survivors was papillary. This is consistent with data from post-Chernobyl studies reporting that more than 90% of radiation-induced thyroid neoplasms were papillary [30]. Mutations in the RET/PTC oncogene have been reported to play an important role in the development of papillary carcinomas after radiation exposure [31].

Only a few studies reported the treatment followed for second malignancies after leukemia. Borgmann et al. indicated that the treatment was personalized and followed the protocols for the specific secondary malignancy [16]. In the studies that reported the treatments followed for second thyroid cancer, most patients underwent either subtotal or total thyroidectomy, while radioactive iodine (RAI) ablation was also used in some cases [12,18,22]. For patients with lymph node involvement, cervical nodal dissection was also performed [18].

### 3.7. Outcomes After Second Primary Thyroid Cancer

The 5-year survival outcome after thyroid cancer was reported in 11 of 18 studies, for a total of 102 of 162 leukemia survivors with second primary thyroid cancer, all of whom were alive at follow-up, resulting in a survival rate of 100%.

When considering all patients with SPMs, Berger et al. found significantly lower survival rates in those with SPMs than in those without SPMs (59.5 ± 7.3% vs. 75.7 ± 0.8% at 5 years; *p* = 0.003) [14]. However, when the authors compared patients with SPMs caused by irradiation and those with SPMs unrelated to irradiation, the former seemed to have better survival rates (82.2 ± 8.1%). The authors attributed this to the higher frequency of papillary thyroid carcinoma as the SPM among these patients, which generally has a good prognosis [14]. In their study, Toret et al. investigated the survival outcomes of patients who developed SPMs after childhood leukemia [28]. The authors reported a mean survival time of 165.9 ± 23.1 months and 5-year survival rates estimated at 70%. Moreover, they found that patients with SPMs who had been diagnosed with ALL at a younger age (<5 years) had better survival outcomes than those diagnosed at a later age [28]. Notably, these findings indicate the pooled survival of all patients with SPMs and not specifically of those with second-to-thyroid cancer.

For childhood cancer survivors, including childhood leukemia survivors, Koh et al. investigated the survival outcomes in those who developed SPMs, stratifying them by the type of SPM [19]. The survival rate was much better for second thyroid cancer than for other second cancers, including AML, osteosarcomas, and central nervous system tumors [19]. Specifically, the overall 5-year survival probability for the 15 patients who developed thyroid cancer was 100%, including two ALL survivors. Consistent results were reported by other studies, including Perkins et al. in a sample of 4806 leukemia survivors among whom 18 developed thyroid cancer [23]; Oudin et al. in smaller sample of 502 patients, among whom 26 had thyroid cancer [22]; and Schiemegelow et al. in a very large sample of around 54,000 patients with ALL, among whom 32 developed thyroid cancer [25]. Gow et al. also reported 100% survival in 17 patients who developed second thyroid cancer, including six patients who had a primary diagnosis of ALL, although for a shorter median follow-up period of 3.6 (0.2–21.7) years [18]. Finally, Martucci et al. investigated the event-free survival of patients with second thyroid cancer, showing similar results to those in patients with primary thyroid cancer [20].

## 4. Discussion

This systematic review aimed to summarize the current evidence on the occurrence of thyroid cancer after childhood leukemia. We found that the crude incidence of second malignancies after childhood leukemia is 10.1 per 1000 patients. Of these, thyroid carcinoma occurred in 1.5 per 1000 survivors, accounting for about 15% of second malignancies in this population. The 5-year survival was 100% in all studies that reported survival outcomes.

Our findings demonstrate that thyroid cancer represents a substantial proportion of second malignancies in childhood leukemia survivors. Previous evidence indicates that thyroid cancer is one of the most common types of SPMs among all childhood cancer survivors, mainly due to exposure during therapy [32]. Given this elevated risk, childhood cancer survivors require systematic surveillance [33]. The current guidelines recommend frequent monitoring for various endocrine disorders in childhood cancer survivors to enable early detection and prompt management, ensuring optimal patient outcomes. For example, the guidelines of the Children’s Oncology Group recommend the palpation of the thyroid gland once every year, while guidelines from other societies recommend ultrasound examination once every 3–5 years [34,35]. The surveillance should be initiated within the first 5 years of any form of radiotherapy, including total body irradiation [35].

Consistent with the established literature [32], a radiation-based regimen for leukemia emerged as an important risk factor in our analysis. Among the included studies in our review, all reported the use of radiotherapy except one that did not provide information on the treatment used for leukemia. Radiation exposure was previously identified as a significant risk factor for thyroid cancer among adults, even when used at low levels for diagnostic purposes [36]. However, results in adult cancer patients are not as strong as in pediatrics. A previous systematic review and meta-analysis indicated no significant risk of thyroid cancer development following radiation therapy in adult patients, although it stressed that more research is needed on the topic [8]. This discrepancy between children and adults is expected because of the higher sensitivity of the pediatric thyroid gland to radiation [37]. Other risk factors for thyroid cancer in childhood leukemia survivors identified via our systematic review included a young age at leukemia diagnosis and female sex, in agreement with a previous pooled analysis of 16,757 childhood cancer survivors [38].

Beyond incidence and risk factors, treatment outcomes of thyroid cancer are encouraging. In our review, the reported treatment approaches generally followed the current guidelines for the management of thyroid cancer, which indicate total thyroidectomy as the preferred option [39]. Thyroidectomy may be followed by the administration of RAI, which may help reduce the incidence of thyroid cancer or treat any persistent thyroid disease. The use of RAI is, however, preserved for patients with high-risk thyroid cancer and is not recommended for patients at low risk [39]. Treatment variations according to the patient presentation were documented in one study, such as nodal dissection in patients who had lymph node involvement [18]. Overall, these treatments have excellent outcomes, especially in early-stage thyroid cancer [40]. In agreement, our systematic review indicated a 100% survival rate for at least 5 years in patients with second thyroid cancer following leukemia.

### Limitations

The study results should be considered in light of certain limitations. The main limitation was the limited number of studies focusing solely on patients with ALL, which did not allow us to calculate the incidence of second thyroid cancer specifically in this cohort but rather in all patients with leukemia, including ALL, AML, CML, and biphenotypic acute leukemia (Table 1). Another limitation is that we only calculated the crude incidence of thyroid cancer and did not conduct a meta-analysis. This was, firstly, because the sample size of leukemia survivors was quite small in some studies (e.g., only two patients had leukemia, both of whom developed thyroid cancer), and second, because only patients with thyroid cancer as an SPM had been included according to the selection criteria in other studies. In both cases, this resulted in an incidence of second thyroid cancer of 100%, which is far from the true occurrence of this SPM in childhood leukemia survivors. Furthermore, we did not conduct a meta-analysis of the survival outcomes. Although such an analysis was possible, we considered it would not have provided any additional information, since the survival rates were 100% in all studies that reported this outcome after thyroid cancer. Finally, since none of the identified studies investigated the prognostic impact of the occurrence of thyroid cancer in childhood leukemia survivors, we could not conclude whether these patients are at risk of developing third cancers or other comorbidities.

## 5. Conclusions

We found that thyroid carcinomas account for ~15% of secondary malignancies after childhood leukemia, including ALL, with a crude incidence of approximately 1.5 per 1000 patients. Radiation exposure remains a significant risk factor despite its overall reduced use for the treatment of leukemia in the last few decades. Although survival rates among these patients remain high (100% according to our analysis), children who have survived leukemia should be regularly monitored for the appearance of second thyroid cancer to ensure prompt treatment. Our study sets the basis for further research to determine the incidence and outcomes of thyroid cancer in childhood ALL survivors and to develop novel, radiation-free therapies to reduce the associated risks. Since our findings are based mainly on the subgroup analyses of various cohort studies, a future meta-analysis of larger cohorts of patients with ALL is necessary. Moreover, while establishing the incidence of thyroid cancer after childhood leukemia is important, it is also necessary to establish the prognostic implications of these malignancies later in the life of these patients. This may help inform care and follow-up. The careful analysis of the associated risk factors might also help prevent the development of these second malignancies.

## Figures and Tables

**Table 1:** Characteristics of the included studies.

Study	Study Setting	Type of Leukemia	Treatment for Leukemia	Sample Size (Total/ Leukemia)	No./Sex of Patients with Second Malignancies		Age at Diagnosis of Leukemia or of Primary Malignancy (Years) ^a^	Time to Thyroid Cancer Diagnosis After Leukemia (Years)	Type of Thyroid Cancer	Treatment for Thyroid Cancer	Reported Outcomes	Survival at 5 Years After Thyroid Cancer Diagnosis
					Total/ After Leukemia	Thyroid Cancer After Leukemia						
Acharya et al., 2003 [12]	USA	ALL and AML	Radiation therapy	33/3	13/3	3 F (2 after ALL and 1 after AML)	5.6 ± 1.0	15.3 ± 3.3	Follicular and papillary	Near-total thyroidectomy and RAI therapy	Thyroid neoplasms after radiation therapy	NR
Bebeshko et al., 2021 [13]	Ukraine	ALL	Standard chemotherapy and radiotherapy	92/92	1/1	1 F	NR	11	Papillary	NR	Incidence of thyroid disease	NR
Berger et al., 2011 [14]	France	ALL and CML	TBI (n = 11), allogeneic marrow transplantation (n = 11)	2907/854	54 (28 F)/21	3 (2 after ALL and 1 after CML)	7.8 ± 5.5	15.0 ± 5.8	NS	NR	Incidence of second malignancy and patient outcomes	3/3
Bhatia et al., 2002 [15]	USA and Canada	ALL	CP (100%), anthracyclines (100%), and radiation (50%)	8831/8831	63/63	4 (2F and 2M)	2.9 (1.5–4.0)	9.7 (5.5–11.8)	Papillary	NR	Risk of developing second malignancy	4/4
Borgman et al., 2008 [16]	Germany	ALL	Cranial irradiation, TBI, multi-drug chemotherapy protocols	1376/1376	21/21	2 (1F and 1M)	5.1 ± 0.7	15.7 ± 6.9	NS	NS (treatment based on disease-specific protocol)	Cumulative incidence of secondary malignancy	2/2
Echecopar et al., 2024 [17]	Spain	ALL	TBI ± HSCT or HSCT alone	57/57	7/7	2	6.3 ± 3.9 ^b^	NR	Follicular	NR	Survival of ALL patients after TBI	NR
Gow et al., 2003 [18]	USA	ALL	Chemotherapy + radiation (70.6%), chemotherapy only (5.9%), radiation only (11.8%)	17/17	17 (13 F)/6	6	NR	16.2 (0.9–29.2)	Papillary (88.2%) and follicular (11.8%)	Total thyroidectomy, cervical nodal dissection, and RAI ablation	Outcomes of thyroid carcinoma	6/6
Koh et al., 2016 [19]	Korea	ALL, AML, BAL	Chemotherapy, TBI, and HSCT	102/18	18/18	2	6.6 (0–19.7) ^c^	6.8 (4.1–18.5) ^c^	Papillary	Thyroidectomy	Outcomes of second malignancy	2/2
Martucci et al., 2022 [20]	Italy, Brazil, UK, Argentina, and France	ALL	Radiotherapy	255/5	13/5	5	14 (1–18.4) ^c^	9.0 ± 4.8	Adenoma and papillary	Hemi- or total thyroidectomy	Survival	5/5
Maule et al., 2007 [21]	Australia, Canada, Sweden, Finland, Denmark, Scotland, Iceland, Norway, Slovenia, Spain, and Singapore	NS	NR	16,540/ 12,731	133/ 67	9	1–14	10–19	NS	NR	Cumulative incidence of thyroid cancer	NR
Oudin et al., 2016 [22]	France	ALL and AML	CNS irradiation, HSCT with or without prior TBI	502/502	26/26	26	7.4 ± 0.2 ^b^	NR Age at diagnosis of TC: 20.5 (17.1–24.1)	Papillary	RAI therapy and subtotal or total thyroidectomy	Prevalence and risk factors for thyroid cancer	26/26
Perkins et al., 2013 [23]	USA	ALL and AML	Radiation therapy	4806/4806	82/82	18 (16 after ALL; 14 F)	7.0 (0–18.0)	~7–35.5	NS	NR	Survival of primary and second malignancy	18/18
Renard et al., 2011 [24]	USA	ALL	Radiotherapy and regimens including topoisomerase-II inhibitors, HSCT	2216/2077	22/20	2 F	5.0 ± 1.0	5.5 ± 0.9	Papillary	NR	Incidence of second malignancy	2/2
Schiemegelow et al., 2013 [25]	North America, Europe, and Asia	ALL	CP, EPT, CNS irradiation	54,058/54,058	642/642	32 (17 F)	5.0 (3.1–6.5)	10.1 (7.8–13.5)	NS	NR	Incidence of second malignancy and survival	32/32
Socie’ et al., 2000 [26]	USA	ALL	Allogeneic BMT, conditioning regimen (TBI or limited field irradiation ± CP ± other drugs), SCT	3182/2022 ^d^	45/45	2 ^d^	7.4 ± 2.6 ^e^	7.7 ± 4.5	Papillary	NR	Risk of developing second malignancy	2/2
Taylor et al., 2009 [27]	UK	ALL and AML	TBI, cranial radiotherapy, chemotherapy	17,980/NS	50 (31 F)/13	13 (9 after ALL and 4 after AML)	2–14	8–33	Papillary, follicular, other	NR	Cumulative incidence of thyroid carcinoma	NR
Toret et al., 2024 [28]	Turkey	ALL	Cranial radiotherapy (39%), HSCT (12%), TBI (12%)	10,360/10,360	41/41	5	5.3 ± 4.3	18.9 ± 8.4 ^f^	NS	NR	Survival	NR
Veiga et al., 2012 [29]	USA and Canada	NS	Chemotherapy (alkylating agents, anthracyclines, bleomycin, platinum compounds, and EPT), radiotherapy	12,547/4261	119/27	27	5.7 ± 4.0	21.0 ± 7.7	Papillary and mixed papillary, follicular, other	NR	Cumulative incidence of thyroid cancer according to the treatment regimen	NR

ALL, acute lymphoblastic leukemia; AML, acute myelogenous leukemia; BAL, biphenotypic acute leukemia; BMT, bone marrow transplantation; CML, chronic myeloid leukemia; CP, cyclophosphamide; EPT, epipodophyllotoxin; HSCT, hematopoietic stem cell transplant; NR, not reported; NS, not specified; RAI, radioactive iodine; SCT, stem cell transplant; TBI, total body irradiation; TC, thyroid cancer. The age at leukemia diagnosis and time to thyroid cancer diagnosis are presented as mean ± standard deviation or as median (IQR or range). These data were collected from each study or calculated using Microsoft Excel according to the provided raw data for individual patients. ^a^ patients with leukemia who developed thyroid cancer unless otherwise specified; ^b^ entire sample, not only to patients with thyroid cancer as SPMs after leukemia; ^c^ entire sample, not only to patients with leukemia; ^d^ includes only cases of ALL and excludes cases of non-lymphocytic leukemia and cases of acute leukemia undifferentiated or not otherwise specified; ^e^ age at BMT; ^f^ latency for diagnosis of all second solid tumors in the sample, not only thyroid cancer.

**Table 2:** Disease and treatment characteristics.

Study	Type of Leukemia	Treatment for Leukemia	Radiation Dose (cGy)	Age at Diagnosis of Leukemia or of Primary Malignancy (Years) ^a^	Time to Thyroid Cancer Diagnosis After Leukemia (Years)	Type of Thyroid Cancer	Treatment for Thyroid Cancer	Survival at 5 Years After Thyroid Cancer Diagnosis
Acharya et al., 2003 [12]	ALL and AML	Radiation therapy	1000–1800	5.6 ± 1.0	15.3 ± 3.3	Follicular and papillary	Near-total thyroidectomy and RAI therapy	NR
Bebeshko et al., 2021 [13]	ALL	Standard chemotherapy and radiotherapy	1200–1800	NR	11	Papillary	NR	NR
Berger et al., 2011 [14]	ALL and CML	TBI and allogeneic marrow transplantation	1200	7.8 ± 5.5	15.0 ± 5.8	Papillary	NR	3/3
Bhatia et al., 2002 [15]	ALL	CP (100%), anthracyclines (100%), and radiation (50%)	1800–2400	2.9 (1.5–4.0)	9.7 (5.5–11.8)	Papillary	NR	4/4
Borgman et al., 2008 [16]	ALL	Cranial irradiation, TBI, multi-drug chemotherapy protocols	Cranial: 1800–2400 TBI: 1200	5.1 ± 0.7	15.7 ± 6.9	NS	NS (treatment based on disease-specific protocol)	2/2
Echecopar et al., 2024 [17]	ALL	TBI ± HSCT or HSCT alone	NR	6.3 ± 3.9 ^b^	NR	Follicular	NR	NR
Gow et al., 2003 [18]	ALL	chemotherapy + radiation (70.6%), chemotherapy only (5.9%), radiation only (11.8%)	NS	NR	16.2 (0.9–29.2)	Papillary (88.2%); follicular (11.8%)	Total thyroidectomy, cervical nodal dissection, and RAI ablation	6/6
Koh et al., 2016 [19]	ALL, AML, BAL	Chemotherapy, TBI, and HSCT	NR	6.6 (0–19.7) ^c^	6.8 (4.1–18.5) ^c^	Papillary	Thyroidectomy	2/2
Martucci et al., 2022 [20]	ALL	Radiotherapy	NR	14 (1–18.4) ^c^	9.0 ± 4.8	Adenoma and papillary	Hemi- or total thyroidectomy	5/5
Maule et al., 2007 [21]	NS	NR	NR	1–14	10–19	NS	NR	NR
Oudin et al., 2016 [22]	ALL and AML	CNS irradiation and HSCT with or without prior TBI	1800 and 2400	7.4 ± 0.2 ^b^	NR Age at diagnosis of TC: 20.5 (17.1–24.1)	Papillary	RAI therapy and subtotal or total thyroidectomy	26/26
Perkins et al., 2013 [23]	ALL and AML	Radiation therapy	NR	7.0 (0–18.0)	~7–35.5	NS	NR	18/18
Renard et al., 2011 [24]	ALL	Radiotherapy and regimens including topoisomerase-II inhibitors and HSCT	NR	5.0 ± 1.0	5.5 ± 0.9	Papillary	NR	2/2
Schiemegelow et al., 2013 [25]	ALL	CP, EPT, CNS irradiation	NR	5.0 (3.1–6.5)	10.1 (7.8–13.5)	NS	NR	32/32
Socie’ et al., 2000 [26]	ALL	Allogeneic BMT, conditioning regimen (TBI or limited field irradiation ± CP ± other drugs), SCT	low dose or high dose (≥1000 single-dose or ≥1300 fractionated)	7.4 ± 2.6 ^d^	7.7 ± 4.5	Papillary	NR	2/2
Taylor et al., 2009 [27]	ALL and AML	TBI, cranial radiotherapy, chemotherapy	Cranial: 1000–2500 TBI: 1000	2–14	8–33	Papillary, follicular, other	NR	NR
Toret et al., 2024 [28]	ALL	Cranial radiotherapy (39%), HSCT (12%), TBI (12%)	NR	5.3 ± 4.3	18.9 ± 8.4 ^e^	NS	NR	NR
Veiga et al., 2012 [29]	NS	Chemotherapy (alkylating agents, anthracyclines, bleomycin, platinum compounds, and EPT) and radiotherapy	0–4000	5.7 ± 4.0	21.0 ± 7.7	Papillary and mixed papillary, follicular, other	NR	NR

ALL, acute lymphoblastic leukemia; AML, acute myelogenous leukemia; BAL, biphenotypic acute leukemia; BMT, bone marrow transplantation; CML, chronic myeloid leukemia; CP, cyclophosphamide; EPT, epipodophyllotoxin; HSCT, hematopoietic stem cell transplant; NR, not reported; NS, not specified; RAI, radioactive iodine; SCT, stem cell transplant; TBI, total body irradiation; TC, thyroid cancer. The age at leukemia diagnosis and time to thyroid cancer diagnosis are presented as mean ± standard deviation or as median (IQR or range). These data were collected from each study or calculated using Microsoft Excel according to the provided raw data for individual patients. ^a^ patients with leukemia who developed thyroid cancer unless otherwise specified; ^b^ entire sample, not only to patients with thyroid cancer as SPMs after leukemia; ^c^ entire sample, not only to patients with leukemia; ^d^ includes only cases of ALL and excludes cases of non-lymphocytic leukemia and cases of acute leukemia undifferentiated or not otherwise specified; ^e^ age at BMT; ^f^ latency for diagnosis of all second solid tumors in the sample, not only thyroid cancer.

**Table 3:** The methodological quality of the included studies.

Study ID (Authors; Reference)	Selection (Maximum of 3 Stars)	Comparability (Maximum of 2 Stars)	Outcomes (Maximum of 3 Stars)	Quality Assessment ^a^
Acharya et al., 2003 [12]	3	1	3	Good
Bebeshko et al., 2021 [13]	2	1	3	Fair
Berger et al., 2011 [14]	3	2	3	Good
Bhatia et al., 2002 [15]	3	1	3	Good
Borgman et al., 2008 [16]	3	1	3	Good
Echecopar et al., 2024 [17]	3	1	3	Good
Gow et al., 2003 [18]	3	1	3	Good
Koh et al., 2016 [19]	3	1	2	Good
Martucci et al., 2022 [20]	3	1	3	Good
Maule et al., 2007 [21]	3	2	3	Good
Oudin et al., 2016 [22]	3	2	3	Good
Perkins et al., 2013 [23]	3	2	3	Good
Renard et al., 2011 [24]	3	1	3	Good
Schiemegelow et al., 2013 [25]	3	2	3	Good
Socie’ et al., 2000 [26]	3	2	3	Good
Taylor et al., 2009 [27]	3	1	3	Good
Toret et al., 2024 [28]	3	1	3	Good
Veiga et al., 2012 [29]	3	2	3	Good

AHRQ, Agency for Healthcare Research and Quality. ^a^ quality assessment as per the AHRQ standard [11].

## Data Availability

The original contributions presented in this study are included in the article.

## Abbreviations

The following abbreviations are used in this manuscript:

ALL	acute lymphoblastic leukemia
AML	acute myelogenous leukemia
CML	chronic myeloid leukemia
IRB	Institutional Review Board
NOS	Newcastle Ottawa Scale
PRISMA	Preferred Reporting Items for Systematic Reviews and Meta-Analyses
RAI	radioactive iodine
SEER	Surveillance, Epidemiology, and End Results
SPM	second primary malignancies
